# Supplementary material for: Complexome profiling of the Chlamydomonas psb28 mutant reveals TEF5 as an early PSII assembly factor
Source: Plant Cell. 2025 Mar 17;37(6):koaf055. doi: 10.1093/plcell/koaf055 (PMC12164590; doi:10.1093/plcell/koaf055)
Supplement: koaf055_Supplementary_Data [file koaf055_supplementary_data.zip › psb28_interactive.zip!/plots/intensity/qualityPlots/peptides/_Cre12.g503650.t1.2.html]

### Cre12.g503650.t1.2

Trivial name:   
SeqCoverage [median]: 25.30  
Mass: 33.45 kDa  
  
 

| Sequence | MissedCleavage | Mass [Da] | wtIntensity | muIntensity | Score |
| --- | --- | --- | --- | --- | --- |
| ATHMAAVPADIDR | 0 | 1366.67 | 0.00 | 1186.89 | 49.48 |
| ATHMAAVPADIDRNR | 1 | 1636.81 | 0.00 | 3089.98 | 68.03 |
| EALNFTAR | 0 | 920.47 | 0.00 | 4553.60 | 106.36 |
| GFSEYEAVVEGDIK | 0 | 1541.72 | 493.17 | 4063.16 | 145.91 |
| SGGKGDDIQER | 1 | 1160.54 | 891.10 | 3926.75 | 102.40 |
| STTSSAGGGAAAYETK | 0 | 1457.66 | 870.83 | 1369.32 | 113.65 |
| STTSSAGGGAAAYETKK | 1 | 1585.76 | 0.00 | 682.17 | 51.03 |
| WLGGYYEDGKPVR | 1 | 1538.75 | 0.00 | 1170.33 | 62.26 |
|  |  |  | 2255.1 | 20042.2 |  |
